# 2-{[(4-{[(2-Hy­droxy­phen­yl)(phen­yl)methyl­idene]amino}­phen­yl)imino](phen­yl)meth­yl}phenol

**DOI:** 10.1107/S1600536811046988

**Published:** 2011-11-09

**Authors:** Anita Blagus, Branko Kaitner

**Affiliations:** a Department of Chemistry, J.J. Strossmayer University, Osijek, Franje Kuhača 20, HR-31000 Osijek, Croatia; b Laboratory of General and Inorganic Chemistry, Department of Chemistry, Faculty of Science, University of Zagreb, Horvatovac 102a, HR-10002 Zagreb, Croatia

## Abstract

The title mol­ecule, C_32_H_24_N_2_O_2_, has a crystallographically imposed inversion centre and exists in the crystal as an enol–imine tautomer. The mol­ecular structure is stabilized by two strong intra­molecular O—H⋯N hydrogen bonds. The dihedral angles between the central benzene ring and the mean planes of the phenyl substituents are 59.99 (1) and 62.79 (2)°. In the crystal, the mol­ecules are arranged into (010) layers *via* C—H⋯π inter­actions.

## Related literature

For general background to Schiff bases, see: Blagus *et al.* (2010 ▶). For similar structures derived from *p*-phenyl­enediamine, see: Al-Douh *et al.* (2009 ▶); Hoshino *et al.* (1988 ▶); Inabe *et al.* (1994 ▶).
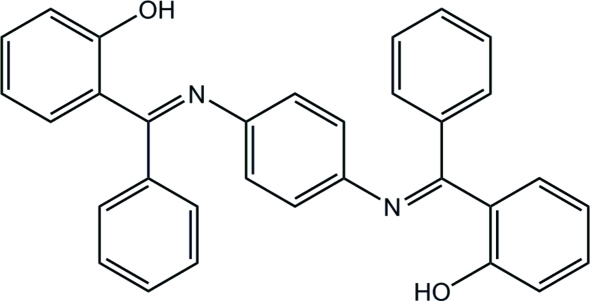


## Experimental

### 

#### Crystal data

C_32_H_24_N_2_O_2_
*M* *_r_* = 468.53
Orthorhombic, 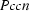

*a* = 17.383 (4) Å
*b* = 14.595 (3) Å
*c* = 9.476 (2) Å
*V* = 2404.1 (9) Å^3^
*Z* = 4
Mo *K*α radiation
μ = 0.08 mm^−1^
*T* = 298 K
0.6 × 0.5 × 0.3 mm

#### Data collection

Oxford Diffraction Xcalibur CCD diffractometer
18421 measured reflections
2361 independent reflections
1559 reflections with *I* > 2σ(*I*)
*R*_int_ = 0.053

#### Refinement

*R*[*F* ^2^ > 2σ(*F* ^2^)] = 0.052
*wR*(*F* ^2^) = 0.149
*S* = 1.03
2361 reflections
166 parameters
H atoms treated by a mixture of independent and constrained refinement
Δρ_max_ = 0.25 e Å^−3^
Δρ_min_ = −0.19 e Å^−3^

### 

Data collection: *CrysAlis CCD* (Oxford Diffraction, 2003 ▶); cell refinement: *CrysAlis RED* (Oxford Diffraction, 2003 ▶); data reduction: *CrysAlis RED*; program(s) used to solve structure: *SHELXS97* (Sheldrick, 2008 ▶); program(s) used to refine structure: *SHELXL97* (Sheldrick, 2008 ▶); molecular graphics: *ORTEP-3* (Farrugia, 1997 ▶); software used to prepare material for publication: *WinGX* (Farrugia, 1999 ▶), *PARST97* (Nardelli, 1995 ▶) and *Mercury* (Macrae *et al.*, 2006 ▶).

## Supplementary Material

Crystal structure: contains datablock(s) I, global. DOI: 10.1107/S1600536811046988/gk2425sup1.cif

Structure factors: contains datablock(s) I. DOI: 10.1107/S1600536811046988/gk2425Isup2.hkl

Supplementary material file. DOI: 10.1107/S1600536811046988/gk2425Isup3.cml

Additional supplementary materials:  crystallographic information; 3D view; checkCIF report

## Figures and Tables

**Table 1:** Hydrogen-bond geometry (Å, °) *Cg* is the centroid of the C1–C6 ring.

*D*—H⋯*A*	*D*—H	H⋯*A*	*D*⋯*A*	*D*—H⋯*A*
O1—H1⋯N1	0.91 (2)	1.73 (2)	2.569 (2)	152 (2)
C15—H15⋯*Cg*^i^	0.93	2.93	3.748 (2)	148

Symmetry code: (i) 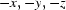.

**Fig. 1.**
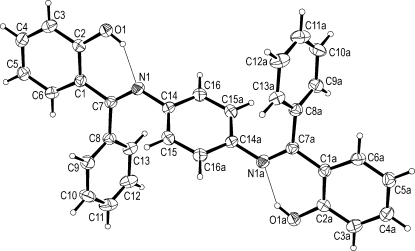
ORTEP view of the title molecule with displacement ellipsoids drawn at the 50% probability level. Label a refers to the atoms with the symmetry code: –x, –y, –z + 1.

**Fig. 2.**
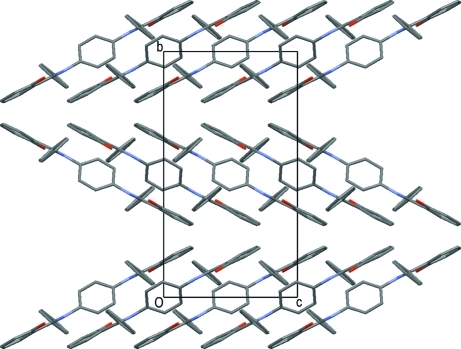
Stacking of (0 1 0) layers along the b-direction. In spite of non-planarity of the title molecule mutual molecular arrangement in neighboring layers corresponds to the herringbone motif typical for fused ring aromatic planar hydrocarbon molecules.

## supplementary crystallographic information

### Comment

There is constant interest in investigation of solid-state structures and properties of Schiff bases and their metal complexes (Blagus *et al.*, 2010 and references therein). The title Schiff base is derived from *p*-phenylenediamine and the structures of three Schiff bases derived from *p*-phenylenediamine and different aldehydes [(i) vanillin (Al-Douh *et al.*, 2009), (ii) salicylaldehyde (Hoshino *et al.*, 1988) and(iii) 2-OH-1-naphthaldehyde (Inabe *et al.*, 1994)] were published since 1988. Two main features that define the shape of the title molecule are: (i) strong intramolecular O–H···N hydrogen bond and (ii) spatial orientation of four terminal aromatic rings with respect to the central one (Fig. 1). The C1–C6 ring and pseudo-aromatic O1–H1–N1–C1–C2–C7 ring are almost co-planar with a dihedral angle of 1.79 (1)° and the displacement of H1 atom from the best plane of pseudo-aromatic ring of 0.016 Å. The bond distances characterizing the enol-imine tautomeric form of (I) are as expected. Taking the central ring C14–C14^i^–C15–C15^i^–C16–C16^i^ [(i): –*x*, –*y*, –*z+*1] as the pivotal one, the interplanar angles between this ring and the rings C1–C6 and C8–C13 are 59.99 (1) and 62.79 (2)°, respectively. The latter rings intersect at an angle of 67.74 (1)°. In crystal packing some weak C–H···π interactions can be observed that organize the molecules into (0 1 0) layers shown in Fig. 2.

### Experimental

The title compound was prepared by the condensation reaction of the aromatic diamine and aromatic 2-OH-ketone in molar ratio 1: 2. Ethanolic solutions of 2-hydroxybenzophenone (10 mmol) and *o*-phenylenediamine (5 mmol) were stirred for 3 h. The resulting brown resinous product was dissolved in ether and overlaid with the same volume of *n*-hexane. After one month, red-brown crystals suitable for single-crystal X-ray analysis were obtained by slow evaporation from the solution. IR spectrum was recorded on Shimadzu FTIR-8400 spectrophotometer cm^-1^: 3378, 1625, 1486, 1335, 1246, 759, 702.

### Refinement

The O—H group hydrogen atom was located in a difference Fourier map and freely refined. The coordinates of H atoms bonded to C were calculated (C–H = 0.96 Å) and these H atoms were refined in a riding model approximation with *U*_iso_(H) = 1.2*U*_eq_ (C).

### Crystal data

**Table:**

C_32_H_24_N_2_O_2_	*F*(000) = 984
*M**_r_* = 468.53	*D*_x_ = 1.294 Mg m^−^^3^
Orthorhombic, *P**c**c**n*	Mo *K*α radiation, λ = 0.71073 Å
Hall symbol: -P 2ab 2ac	Cell parameters from 2361 reflections
*a* = 17.383 (4) Å	θ = 4–26°
*b* = 14.595 (3) Å	µ = 0.08 mm^−^^1^
*c* = 9.476 (2) Å	*T* = 298 K
*V* = 2404.1 (9) Å^3^	Prism, red-brown
*Z* = 4	0.6 × 0.5 × 0.3 mm

### Data collection

**Table:**

Oxford Diffraction Xcalibur CCD diffractometer	1559 reflections with *I* > 2σ(*I*)
Radiation source: fine-focus sealed tube	*R*_int_ = 0.053
graphite	θ_max_ = 26.0°, θ_min_ = 3.8°
ω scan	*h* = −21→21
18421 measured reflections	*k* = −18→15
2361 independent reflections	*l* = −11→11

### Refinement

**Table:**

Refinement on *F*^2^	Primary atom site location: structure-invariant direct methods
Least-squares matrix: full	Secondary atom site location: difference Fourier map
*R*[*F*^2^ > 2σ(*F*^2^)] = 0.052	Hydrogen site location: inferred from neighbouring sites
*wR*(*F*^2^) = 0.149	H atoms treated by a mixture of independent and constrained refinement
*S* = 1.03	*w* = 1/[σ^2^(*F*_o_^2^) + (0.0869*P*)^2^] where *P* = (*F*_o_^2^ + 2*F*_c_^2^)/3
2361 reflections	(Δ/σ)_max_ < 0.001
166 parameters	Δρ_max_ = 0.25 e Å^−^^3^
0 restraints	Δρ_min_ = −0.19 e Å^−^^3^

### Special details

**Table:**

Geometry. All e.s.d.'s (except the e.s.d. in the dihedral angle between two l.s. planes) are estimated using the full covariance matrix. The cell e.s.d.'s are taken into account individually in the estimation of e.s.d.'s in distances, angles and torsion angles; correlations between e.s.d.'s in cell parameters are only used when they are defined by crystal symmetry. An approximate (isotropic) treatment of cell e.s.d.'s is used for estimating e.s.d.'s involving l.s. planes.
Refinement. Refinement of *F*^2^ against ALL reflections. The weighted *R*-factor *wR* and goodness of fit *S* are based on *F*^2^, conventional *R*-factors *R* are based on *F*, with *F* set to zero for negative *F*^2^. The threshold expression of *F*^2^ > σ(*F*^2^) is used only for calculating *R*-factors(gt) *etc*. and is not relevant to the choice of reflections for refinement. *R*-factors based on *F*^2^ are statistically about twice as large as those based on *F*, and *R*- factors based on ALL data will be even larger.

### Fractional atomic coordinates and isotropic or equivalent isotropic displacement parameters (Å^2^)

**Table:**

	*x*	*y*	*z*	*U*_iso_*/*U*_eq_	
O1	0.10687 (9)	0.20165 (9)	0.10710 (16)	0.0382 (4)	
H1	0.0915 (13)	0.1651 (16)	0.179 (3)	0.046*	
N1	0.07823 (10)	0.05834 (11)	0.25539 (18)	0.0307 (4)	
C1	0.13856 (11)	0.04766 (13)	0.0306 (2)	0.0291 (5)	
C2	0.13707 (12)	0.14397 (13)	0.0114 (2)	0.0301 (5)	
C3	0.16724 (13)	0.18187 (14)	−0.1114 (2)	0.0350 (5)	
H3	0.1650	0.2449	−0.1249	0.042*	
C4	0.20034 (12)	0.12759 (14)	−0.2129 (2)	0.0362 (5)	
H4	0.2204	0.1542	−0.2941	0.043*	
C5	0.20404 (12)	0.03367 (14)	−0.1950 (2)	0.0350 (5)	
H5	0.2269	−0.0030	−0.2636	0.042*	
C6	0.17364 (12)	−0.00509 (14)	−0.0750 (2)	0.0328 (5)	
H6	0.1764	−0.0683	−0.0636	0.039*	
C7	0.10431 (12)	0.00524 (13)	0.1571 (2)	0.0290 (5)	
C8	0.10237 (12)	−0.09729 (13)	0.1671 (2)	0.0294 (5)	
C9	0.05715 (13)	−0.14864 (14)	0.0762 (2)	0.0363 (6)	
H9	0.0289	−0.1196	0.0059	0.044*	
C10	0.05388 (14)	−0.24324 (15)	0.0898 (3)	0.0417 (6)	
H10	0.0221	−0.2771	0.0306	0.050*	
C11	0.09736 (14)	−0.28720 (15)	0.1902 (3)	0.0453 (6)	
H11	0.0961	−0.3507	0.1973	0.054*	
C12	0.14278 (13)	−0.23687 (14)	0.2802 (3)	0.0441 (6)	
H12	0.1723	−0.2666	0.3481	0.053*	
C13	0.14491 (12)	−0.14183 (14)	0.2704 (2)	0.0377 (6)	
H13	0.1748	−0.1081	0.3330	0.045*	
C14	0.03947 (12)	0.02512 (13)	0.3774 (2)	0.0289 (5)	
C15	−0.02406 (13)	−0.03231 (13)	0.3698 (2)	0.0319 (5)	
H15	−0.0407	−0.0539	0.2826	0.038*	
C16	−0.06282 (12)	−0.05766 (14)	0.4913 (2)	0.0318 (5)	
H16	−0.1049	−0.0968	0.4851	0.038*	

### Atomic displacement parameters (Å^2^)

**Table:**

	*U*^11^	*U*^22^	*U*^33^	*U*^12^	*U*^13^	*U*^23^
O1	0.0542 (10)	0.0276 (8)	0.0328 (9)	−0.0031 (7)	0.0041 (8)	0.0007 (7)
N1	0.0337 (10)	0.0296 (9)	0.0288 (10)	−0.0006 (8)	0.0005 (8)	0.0031 (8)
C1	0.0284 (11)	0.0310 (11)	0.0279 (12)	−0.0018 (9)	−0.0031 (9)	0.0021 (9)
C2	0.0324 (12)	0.0286 (11)	0.0293 (12)	−0.0027 (9)	−0.0026 (10)	−0.0028 (9)
C3	0.0397 (13)	0.0290 (11)	0.0364 (13)	−0.0025 (10)	−0.0036 (11)	0.0063 (10)
C4	0.0352 (12)	0.0408 (13)	0.0325 (12)	−0.0067 (10)	−0.0001 (10)	0.0037 (10)
C5	0.0341 (12)	0.0384 (13)	0.0326 (13)	0.0039 (10)	0.0028 (10)	0.0007 (10)
C6	0.0340 (12)	0.0276 (11)	0.0369 (13)	0.0001 (9)	−0.0005 (10)	0.0011 (9)
C7	0.0288 (11)	0.0291 (11)	0.0292 (12)	0.0000 (9)	−0.0036 (9)	0.0020 (9)
C8	0.0299 (11)	0.0267 (11)	0.0315 (11)	0.0008 (9)	0.0051 (10)	0.0022 (9)
C9	0.0388 (13)	0.0335 (12)	0.0367 (13)	−0.0012 (10)	−0.0004 (11)	−0.0001 (10)
C10	0.0495 (15)	0.0310 (12)	0.0444 (15)	−0.0087 (11)	0.0037 (12)	−0.0065 (11)
C11	0.0507 (15)	0.0260 (11)	0.0592 (17)	−0.0021 (11)	0.0072 (13)	0.0019 (11)
C12	0.0421 (14)	0.0341 (13)	0.0561 (16)	0.0024 (10)	−0.0005 (12)	0.0136 (11)
C13	0.0374 (13)	0.0343 (12)	0.0413 (13)	−0.0050 (10)	−0.0003 (11)	0.0060 (10)
C14	0.0346 (12)	0.0231 (10)	0.0289 (12)	0.0032 (9)	0.0024 (9)	0.0027 (9)
C15	0.0368 (13)	0.0303 (11)	0.0286 (12)	0.0017 (10)	−0.0014 (10)	−0.0020 (9)
C16	0.0317 (12)	0.0283 (11)	0.0353 (13)	−0.0026 (9)	0.0012 (10)	0.0005 (9)

### Geometric parameters (Å, °)

**Table:**

O1—C2	1.344 (2)	C8—C13	1.388 (3)
O1—H1	0.91 (2)	C9—C10	1.388 (3)
N1—C7	1.294 (3)	C9—H9	0.9300
N1—C14	1.423 (3)	C10—C11	1.374 (3)
C1—C6	1.402 (3)	C10—H10	0.9300
C1—C2	1.418 (3)	C11—C12	1.375 (3)
C1—C7	1.474 (3)	C11—H11	0.9300
C2—C3	1.391 (3)	C12—C13	1.391 (3)
C3—C4	1.372 (3)	C12—H12	0.9300
C3—H3	0.9300	C13—H13	0.9300
C4—C5	1.383 (3)	C14—C15	1.388 (3)
C4—H4	0.9300	C14—C16^i^	1.392 (3)
C5—C6	1.376 (3)	C15—C16	1.384 (3)
C5—H5	0.9300	C15—H15	0.9300
C6—H6	0.9300	C16—C14^i^	1.392 (3)
C7—C8	1.500 (3)	C16—H16	0.9300
C8—C9	1.386 (3)		
			
C2—O1—H1	104.8 (15)	C13—C8—C7	119.99 (19)
C7—N1—C14	123.14 (17)	C8—C9—C10	120.2 (2)
C6—C1—C2	117.41 (18)	C8—C9—H9	119.9
C6—C1—C7	121.68 (18)	C10—C9—H9	119.9
C2—C1—C7	120.90 (18)	C11—C10—C9	120.4 (2)
O1—C2—C3	117.56 (18)	C11—C10—H10	119.8
O1—C2—C1	122.76 (18)	C9—C10—H10	119.8
C3—C2—C1	119.67 (19)	C10—C11—C12	119.7 (2)
C4—C3—C2	120.98 (19)	C10—C11—H11	120.1
C4—C3—H3	119.5	C12—C11—H11	120.1
C2—C3—H3	119.5	C11—C12—C13	120.4 (2)
C3—C4—C5	120.4 (2)	C11—C12—H12	119.8
C3—C4—H4	119.8	C13—C12—H12	119.8
C5—C4—H4	119.8	C8—C13—C12	120.0 (2)
C6—C5—C4	119.4 (2)	C8—C13—H13	120.0
C6—C5—H5	120.3	C12—C13—H13	120.0
C4—C5—H5	120.3	C15—C14—C16^i^	118.97 (18)
C5—C6—C1	122.10 (19)	C15—C14—N1	122.68 (18)
C5—C6—H6	118.9	C16^i^—C14—N1	118.15 (18)
C1—C6—H6	118.9	C16—C15—C14	120.35 (19)
N1—C7—C1	118.37 (18)	C16—C15—H15	119.8
N1—C7—C8	122.98 (18)	C14—C15—H15	119.8
C1—C7—C8	118.65 (17)	C15—C16—C14^i^	120.67 (19)
C9—C8—C13	119.14 (19)	C15—C16—H16	119.7
C9—C8—C7	120.86 (18)	C14^i^—C16—H16	119.7
			
C6—C1—C2—O1	−178.21 (19)	N1—C7—C8—C9	114.0 (2)
C7—C1—C2—O1	1.9 (3)	C1—C7—C8—C9	−67.0 (3)
C6—C1—C2—C3	2.3 (3)	N1—C7—C8—C13	−64.5 (3)
C7—C1—C2—C3	−177.61 (19)	C1—C7—C8—C13	114.5 (2)
O1—C2—C3—C4	178.80 (19)	C13—C8—C9—C10	0.8 (3)
C1—C2—C3—C4	−1.7 (3)	C7—C8—C9—C10	−177.8 (2)
C2—C3—C4—C5	0.2 (3)	C8—C9—C10—C11	−2.2 (3)
C3—C4—C5—C6	0.5 (3)	C9—C10—C11—C12	1.8 (4)
C4—C5—C6—C1	0.2 (3)	C10—C11—C12—C13	0.1 (4)
C2—C1—C6—C5	−1.6 (3)	C9—C8—C13—C12	1.0 (3)
C7—C1—C6—C5	178.34 (19)	C7—C8—C13—C12	179.6 (2)
C14—N1—C7—C1	175.68 (17)	C11—C12—C13—C8	−1.5 (3)
C14—N1—C7—C8	−5.3 (3)	C7—N1—C14—C15	−53.9 (3)
C6—C1—C7—N1	174.74 (19)	C7—N1—C14—C16^i^	131.3 (2)
C2—C1—C7—N1	−5.3 (3)	C16^i^—C14—C15—C16	−0.9 (3)
C6—C1—C7—C8	−4.3 (3)	N1—C14—C15—C16	−175.61 (18)
C2—C1—C7—C8	175.60 (19)	C14—C15—C16—C14^i^	0.9 (3)

Symmetry codes: (i) −*x*, −*y*, −*z*+1.

### Hydrogen-bond geometry (Å, °)

**Table:**

Cg is the centroid of the C1–C6 ring.

**Table:**

*D*—H···*A*	*D*—H	H···*A*	*D*···*A*	*D*—H···*A*
O1—H1···N1	0.91 (2)	1.73 (2)	2.569 (2)	152 (2)
C15—H15···Cg^ii^	0.93	2.93	3.748 (2)	148

Symmetry codes: (ii) −*x*, −*y*, −*z*.
